# Span of regularization for solution of inverse problems with application to magnetic resonance relaxometry of the brain

**DOI:** 10.1038/s41598-022-22739-3

**Published:** 2022-11-23

**Authors:** Chuan Bi, M. Yvonne Ou, Mustapha Bouhrara, Richard G. Spencer

**Affiliations:** 1 Department of Psychiatry, University of Maryland, Baltimore, Baltimore, MD 21201 USA; 2 Department of Mathematical Sciences, University of Delaware, Newark, DE 19716 USA; 3 National Institute on Aging, National Institutes of Health, Baltimore, MD 21224 USA

**Keywords:** Applied mathematics, Computational science

## Abstract

We present a new regularization method for the solution of the Fredholm integral equation (FIE) of the first kind, in which we incorporate solutions corresponding to a range of Tikhonov regularizers into the end result. This method identifies solutions within a much larger function space, spanned by this set of regularized solutions, than is available to conventional regularization methods. An additional key development is the use of dictionary functions derived from noise-corrupted inversion of the discretized FIE. In effect, we combine the stability of solutions with greater degrees of regularization with the resolution of those that are less regularized. The span of regularizations (SpanReg) method may be widely applicable throughout the field of inverse problems.

## Introduction

### Regularization of inverse problems arising from the Fredholm equation of the first kind with noisy data

A large class of inverse problems arise from a discretized Fredholm integral equation (FIE) of the first kind. With the introduction of upper and lower bounds based on prior knowledge, this may be written as

1 $$\begin{aligned} y(t) = \int _{\tau _{L}}^{\tau _{U}} A(t,\tau ) f(\tau ) d\tau {=: {\mathscr {A}}f}, \end{aligned}$$

where *y*(*t*) is the recorded signal, $A(t,\tau )$ is called the *kernel* and $f(\tau )$ is the distribution function (DF) to be determined. A discretization of Eq. (1) leads to

2 $$\begin{aligned} y(t_i) = \sum _{j = 1}^n A(t_i, \tau _j) f(\tau _j) \Delta \tau ,\quad i = 1,2,\cdots , m \end{aligned}$$

and

3 $$\begin{aligned} \mathbf {y}= \mathbf {A}\mathbf {f},\end{aligned}$$

where $\mathbf {y}\in \mathbb {R}^m$, $\mathbf {A}\in \mathbb {R}^{m\times n}$ with $A_{i,j} := A(t_i, \tau _j)\Delta \tau$, and $\mathbf {f}\in \mathbb {R}^n$. Here we assume a uniform discretization $\Delta \tau :=\frac{\tau _{L}-\tau _U}{n}$ and $\tau _i=\tau _L+(i-1)\Delta \tau$ for convenience. Depending on the application, soft or hard constraints on regularity of $f(\tau )$ may include, among others, degree of smoothness, $L^{2}$ norm, or total variation^1,2^

One application of this formulation that is of ongoing interest is to magnetic resonance relaxometry (MRR), which estimates the distribution of relaxation times $T_2$ within a sample. Physically, $T_2$, also called the transverse relaxation time, represents the time constant for decay of transverse magnetization. The range of possible $T_2$ values depends on the sample or tissue under study, but typical values may range from $\sim$10 ms to 2000 ms in biomedical studies, with smaller values corresponding to more solid or rigid tissues. Shorter values require specialized techniques to detect, depending on available hardware and experimental protocols. The distribution function $f(T_2)$ of such values can be of great importance in characterizing the physical properties and the material composition of a sample. In MRR, the kernel function is of the special form $A(t,T_2) = \exp (-\frac{t}{T_2})$ and the Fredholm integral equation in (1) is a Laplace transform. Explicitly, the signal model is

4 $$\begin{aligned} y(t) = \int e^{-\frac{t}{T_2}} f(T_2)d T_2. \end{aligned}$$

We emphasize that this serves as a paradigm for a much larger class of physical phenomena, both within magnetic resonance studies and otherwise, leading to essentially identical mathematical considerations.

Equation (4) defines the integrated signal from an ensemble of decaying exponentials, with the contribution for given values of the decay constant $T_2$ determined by $f(T_2)$. Thus, as *t* increases, the overall signal decays into the noise with a mixture of time constants, the distribution of which is to be determined. Practically, the observed data $\mathbf {y}_{\text {ob}}\in \mathbb {R}^m$ is obtained at measurement times $\{t_i\}_{i=1}^m$, and the goal is to determine the $T_2$ distribution $\mathbf {f}\in \mathbb {R}^n$ as defined by the discretized problem:

5 $$\begin{aligned} \mathbf {y}_{\text {ob}}= \mathbf {A}\mathbf {f}+ {\varvec{\omega }}, \quad \mathbf {f}\ge \mathbf {0}, \end{aligned}$$

where $\mathbf {A}\in \mathbb {R}^{m\times n}$ is the kernel matrix with entries $A_{i,j} = e^{-\frac{t_i}{T_{2,j}}} \Delta T_2$ and ${\varvec{\omega }}$ is additive random noise. We define the SNR of the signal by

6 $$\begin{aligned} \text {SNR} = \frac{\max \left| \mathbf {y}_{\text {ob}}\right| }{\text {RMS}({\varvec{\omega }})}, \end{aligned}$$

where RMS$({\varvec{\omega }})$ is the root mean square noise amplitude. The positivity constraint $\mathbf {f}\ge \mathbf {0}$ arises from the physical requirement that $\mathbf {f}$ represents the volume fraction of materials in the sample. The matrix $\mathbf {A}$ inherits the smoothing property of the integral operator in (1), and can exhibit a condition number that is so large that any direct inversion of the noisy data $\mathbf {y}_{\text{ob}}$ without regularization, such as through non-negative least squares (NNLS) analysis^3^, can be extremely unstable.

From the perspective of inverse problems, determination of the discretized $T_2$ distribution $\mathbf {f}$ can be seen as a version of the inverse Laplace transform (ILT), a classic ill-posed problem. An important method for addressing the numerical instability inherent in this process is through Tikhonov regularization. In the context of NNLS, where $\mathbf {f}$ is everywhere non-negative, the native problem

7 $$\begin{aligned} \mathbf {f}_{LS} = \mathop {\hbox {argmin}}_{\hat{\mathbf {f}}\ge \mathbf {0}}\left\Vert \mathbf {A}\hat{\mathbf {f}}- \mathbf {y}_{\text {ob}}\right\Vert _2^2, \end{aligned}$$

is replaced by the closely related problem

8 $$\begin{aligned} \mathbf {f}_{\lambda } = \mathop {\hbox {argmin}}_{\hat{\mathbf {f}}\ge \mathbf {0}} \left\{ \left\Vert \mathbf {A}\hat{\mathbf {f}}- \mathbf {y}_{\text {ob}}\right\Vert _2^2 + \lambda ^2\left\Vert \hat{\mathbf {f}}\right\Vert _2^2 \right\} . \end{aligned}$$

The second term serves to penalize large values of the norm of the recovered DF, limiting sensitivity to noise. The regularization parameter $\lambda$ acts to titrate the relative importance of the two terms in Eq. (8). The recovered DF $\mathbf {f}_{\lambda }$ may be highly dependent on this parameter; selection of an optimal $\lambda$ remains the topic of active research. This formulation in effect replaces the original ill-posed problem, Eq. (7), by Eq. (8), a more well-behaved but different problem^2^. Additionally, prior application-dependent assumptions regarding $\hat{\mathbf {f}}$, such as sparsity or smoothness, can be introduced into the minimization by introduction of suitable alternative or additional regularization terms^3,4^.

### Methods of parameter selection

The problem of selecting $\lambda$ in Tikhonov regularization has been studied for decades, with no universal approach having been identified. Classical methods such as the Morozov discrepancy principle (DP)^5^, the L-curve^6–9^, and generalized cross-validation (GCV)^10–12^ define criteria by which to select $\lambda$. For example, the DP seeks a value of $\lambda$ such that the size of the first term in Eq. (8), called the residual, is matched to the noise level in the observed data. The L-curve method identifies a value of $\lambda$ that defines the corner of the L-shaped curve defined by the log-log plot of the solution norm against the norm of the residual. GCV selects lambda using an expression based on leave-one-out cross-validation. All of these methods seek to identify one optimal $\lambda$ and its corresponding regularized solution, and discard all other solutions. Variations on this include the elastic net, which incorporates two different $\lambda$’s serving what are essentially distinct roles, but still identifies single optimal values for each^13^. A survey of methods can be found in^14^.

The central theme of the present work is that discarding all results except the reconstruction corresponding to a single selected $\lambda$ leads to loss of information that could contribute to the accurate recovery of the DF, $\mathbf {f}$. We have observed that the effects of regularization, for example, variations of widths, amplitudes, and shapes of recovered distributions $\mathbf {f}_{\lambda _j}$, depend on the underlying distribution $\mathbf {f}$. This motivates the notion that the solutions corresponding to values of $\lambda$ other than the one selected may contain additional information, so that improved results may be obtained by incorporating these solutions into the final determination of $\mathbf {f}$. Accordingly, we describe a new method, termed *span of regularization, or SpanReg*, for which the recovered distribution function is a linear combination of regularized solutions across a range of $\lambda$’s. This expands the space of functions from which the desired DF is drawn.

### Gaussian mixture representation and application to determining $T_2$ distribution functions

We use a Gaussian mixture representation as a dictionary to describe the unknown DF, $\mathbf {f}$, as required for our analysis. One approach would be to write the DF as the linear combination of a finite set of Gaussian functions $g_i(\tau ):=g(\tau ;\mu _i,\sigma _i)$, with $\mu _i$ and $\sigma _i$ representing the unknown mean and standard deviation (SD) of a given element of that set. The discretization of the $g_i$’s along the abscissa follows from the discretization of $\mathbf {f}$ along the abscissa, that is, the choice of the set of abscissa $T_2$ values^15^. With a prior assumption of the number *M* of Gaussian components required for an adequate description, the determination of $\mathbf {f}$ can be recast as the non-linear least squares problem:

$$\begin{aligned} \mathop {\hbox {argmin}}_{\left\{ \mu _i, \sigma _i\right\} }{\left\Vert \mathbf {y}_{\text {ob}}- \mathbf {A}\sum _{i=1}^M \mathbf {g}(\tau ;\mu _i, \sigma _i) \right\Vert _2^2}. \end{aligned}$$

Alternatively, by establishing a dictionary of Gaussian functions of specified $\mu _i$ and $\sigma _i$^16,17^ and incorporating the non-negativity of $\mathbf {f}$, we instead have the problem:

$$\begin{aligned} \mathop {\hbox {argmin}}_{\mathbf {c}\ge \mathbf {0}, \sum _i c_i = 1}{\left\Vert \mathbf {y}_{\text {ob}}- \left( \mathbf {A}\mathbf {G}\right) \mathbf {c}\right\Vert _2^2}, \end{aligned}$$

where $G_{jk}=g_k(\tau _j)$, i.e. the columns of the matrix $\mathbf {G}$, $\left\{ \mathbf {g}_i \right\}$, represent an element of the dictionary and $\mathbf {c}$ is the vector of coefficients respectively assigned to these elements. The first approach above has the advantage of significantly reducing the effective dimensionality of the problem^18^, but requires the solution of the highly nonlinear problem of determining the 2*M* variables $\mu _i$ and $\sigma _i$. On the other hand, the second approach requires only sufficient knowledge of the system under study to permit a reasonable selection of dictionary functions; nonlinearity is introduced through the positivity constraint. This problem becomes increasingly ill-posed with increasing *M*, and will in general be severely ill-posed due both to the required value of *M* and the non-orthogonality of the Gaussian dictionary functions.

### Motivation for SpanReg

Because of the ill-posedness of the problem under consideration, regularization is required for constructing $\mathbf {f}$ from $\mathbf {y}_{\text {ob}}$. The goal of SpanReg is to provide an inversion scheme that is more robust to noise as compared with conventional methods and to address the difficulty of choosing the ”best” regularization parameter by simultaneously incorporating different levels of regularization into the reconstruction scheme. We consider Tikhonov regularization with parameter $\lambda$ and denote by ${\mathscr {A}}^{-1}_{\lambda }$ the operator mapping observations to a regularized inverse solution to the linear problem $y_{ob}(t)={\mathscr {A}}f+\omega$ with regularization parameter $\lambda$; this is the well-known pseudoinverse^19^. Note that our problem of interest is constrained, so that the corresponding operator mapping data to regularized solutions is not linear. However, the linear example serves well to illustrate the underlying idea of SpanReg as follows.

For $f(\tau )=\sum _{k=1}^M c_k g_k(\tau )$, given the data and the linear inversion operator ${\mathscr {A}}^{-1}_\lambda$, we have

$$\begin{aligned} {\mathscr {A}}^{-1}_\lambda y_{ob}= & {} {\mathscr {A}}^{-1}_\lambda \left( \sum _{k=1}^M c_k {\mathscr {A}}g_k +\omega \right) = {\mathscr {A}}^{-1}_\lambda \left( \sum _{k=1}^M c_k ({\mathscr {A}}g_k +\omega )-\sum _{k=1}^M c_k\omega +\omega \right) \end{aligned}$$

Imposing the constraint $\sum _{k=1}^M c_k=1$ results in

9 $$\begin{aligned} {\mathscr {A}}^{-1}_\lambda y_{ob}=\sum _{k=1}^M c_k\left\{ {\mathscr {A}}^{-1}_\lambda \left( {\mathscr {A}}g_k+\omega \right) \right\} \end{aligned}$$

Therefore, if the noise $\omega$ were known, then the solution $\{c_k\}_{k=1}^M$ could be recovered by finding what are in effect the coordinates of the inversion of the noise-corrupted data $y_{ob}$ with respect to the noise-corrupted basis $\{{\mathscr {A}}^{-1}_\lambda \left( {\mathscr {A}}g_k+\omega \right) \}_{k=1}^M$. Because the noise $\omega$ in experimental data is unknown, we instead form the ensemble average on both side of (9) to obtain

10 $$\begin{aligned} \langle {\mathscr {A}}^{-1}_\lambda y_{ob} \rangle =\sum _{k=1}^M c_k \bigg \langle \left\{ {\mathscr {A}}^{-1}_\lambda \left( {\mathscr {A}}g_k+\omega \right) \right\} \bigg \rangle . \end{aligned}$$

If the observation $y_{ob}$ could be repeated a sufficient number of times, then the ensemble average of the recovered results will be within the span of these noise-corrupted basis functions. In actual practice, data is generally obtained from a single or from a small number of observations; SpanReg attempts to find within this span the solution that is closest to the underlying distribution in the least square sense, corresponding an optimal set $\{c_k\}$.

We now discuss the artificial case in which $\mathbf {f}\ge \mathbf {0}$ is known, from which a noise-corrupted signal $\mathbf {y}_{\text {ob}}= \mathbf {A}\mathbf {f}+ {\varvec{\omega }}$ can be created; we can determine $\mathbf {f}_{\lambda _j}$ for specific values of $\lambda _{j}$ within a set of regularization parameters $\Lambda$ by solving Eq. (8). As indicated above, the conventional methods for selection of $\lambda$ in effect evaluate results for several $\lambda$’s and retain only the one corresponding to an optimal value, discarding the others. In contrast, we define our approximation of $\mathbf {f}$ as a linear combination of regularized solutions:

$$\begin{aligned} \mathbf {f}\approx \sum _{j=1}^N \alpha _j \mathbf {f}_{\lambda _j} \end{aligned}$$

where $\left\{ \alpha _j \right\} _1^N$ is the solution to the least squares problem

11 $$\begin{aligned} \alpha =\left( \alpha _1,\alpha _2,\ldots ,\alpha _N \right) = \mathop {\hbox {argmin}}_{\alpha \ge \mathbf {0}} \left\Vert \mathbf {f}- \sum _{j=1}^N \alpha _j \mathbf {f}_{\lambda _j}\right\Vert _2^2. \end{aligned}$$

When $\mathbf {f}$ is known, the set of $\alpha _j$’s defined in Eq. (11), with each $\alpha _j$ corresponding to an element of a linear combination of regularized solutions, can always be selected to provide an improvement in signal reconstruction as compared to selection of any single value of lambda. This motivates the SpanReg approach for reconstructing an unknown distribution. However, an algorithm for selecting the best set $\left\{ \alpha _j \right\} _{{j=}1}^N$ in the approximation:

12 $$\begin{aligned} \mathbf {f}\approx \sum _{j=1}^N \alpha _j \mathbf {f}_{\lambda _j} =: \mathbf {f}_{\alpha },\quad \text {where } \alpha _j \ge 0, \end{aligned}$$

for an unknown DF, $\mathbf {f}$, remains to be developed.

An additional relationship arises from the representation of $\mathbf {f}$ as a linear combination of Gaussian dictionary functions $\left\{ \mathbf {g}_i \right\} _{{i=}1}^M$, which we impose with a non-negativity constraint on the expansion coefficients:

13 $$\begin{aligned} \mathbf {f}\approx \sum _{i = 1}^{M} c_i\mathbf {g}_i =: \mathbf {f}_{\mathbf {c}},\quad \text {where } c_i \ge 0. \end{aligned}$$

We consider the case in which the integral over $\mathbf {f}$ equals 1, so that it represents a probability distribution function (PDF). Since $\int g\left( \sigma _i, \mu _i\right) d\tau = 1$, we have the requirement that $\sum c_i = 1$. The detailed implementation of SpanReg is presented in the “Materials and methods” section.

## Results

### Applications of SpanReg to one-dimensional magnetic resonance relaxometry

We illustrate the application of SpanReg to the inverse problem of MRR for transverse relaxation in one dimension, whose discretized version is described in Eq. (5). The objective is to approximate the distribution of transverse relaxation times $T_2$ within a sample from the signal $\mathbf {y}_{ob}$. In the context of our formalism, this distribution function takes the role of $\mathbf {f}$. $T_2$ values will be expressed in milliseconds (ms).

### Comparison of SpanReg and classical parameter selection methods

We evaluated the performance of SpanReg for the reconstruction of a range of DFs consisting of two Gaussian functions with means $\left( \mu _1, \mu _2\right)$ and standard deviations $\left( \sigma _1, \sigma _2 \right) :$

14 $$\begin{aligned} f_{\text {sim}}(T_2; \{\mu _1, \mu _2, \sigma _1, \sigma _2\}) = \frac{1}{\sqrt{2\pi \sigma _1^2}}e^{-\frac{(T_2 - \mu _1)^2}{2\sigma _1^2}} + \frac{1}{\sqrt{2\pi \sigma _2^2}} e^{-\frac{(T_2 - \mu _2)^2}{2\sigma _2^2}}. \end{aligned}$$

**Figure 1:**
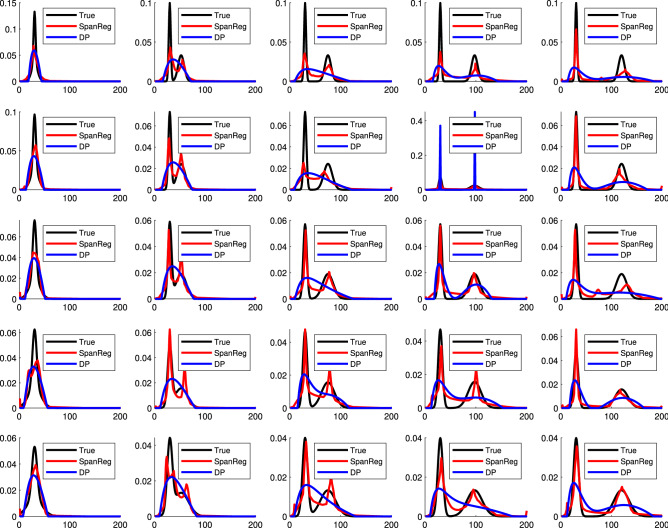
Comparison of the recovered distributions from SpanReg (red) and the discrepancy principle (blue) with the true underlying distributions (black). Each panel represents a $T_2$ distribution consisting of the sum of two Gaussian functions as in Eq. (14). The value of $\sigma _1$, the standard deviation of the left-most component, increases from 2 ms (top row) to 5 ms (bottom row) with $\sigma _2=3\sigma _1$ throughout. Across each row, the ratio of peak separation (RPS) increases from 1 (left-most column) to 4 (right-most column), with the left-most distribution centered at $\mu = 35$ ms.

We examine DF’s with $\sigma _1$ taking values in the set $\{2+0.75k,\, k=0,1,2,3,4\}$ (ms) and $\sigma _2=3\sigma _1$. The separation between the Gaussians is defined by their ratio of peak separation (RPS), defined as $RPS =\frac{\mu _2}{\mu _1}$. We evaluate DF’s with $RPS=1+0.75k$, $k=0,1,2,3,4$ for each $(\sigma _1, \sigma _2)$ pair. None of the Gaussian functions in these distributions is included in the Gaussian dictionary $\left\{ \mathbf {g}_i \right\}$ to avoid trivial solutions. We compare the recovery of each of the 25 resulting DF’s (Fig. 1) obtained from SpanReg and from the DP; here and elsewhere, by DP we indicate NNLS using the DP to set the Tikhonov regularization parameter. This is a particularly suitable comparison since for both methods, the noise level in the data must be known or estimated. As noted, the noise level can be estimated with good accuracy in the case of the superposition of exponentially decaying signals such as in an MRR experiment. The discretization along the $T_2$ axis is defined as above, with $n = 200$ evenly spaced values within the range [1, 200] ms. The measurement time vector is defined as $m=150$ sampling times evenly distributed in $t\in [0.3,400]$ ms. The additive noise level is set to result in SNR of 500. The range of $\lambda$’s used for Tikhonov regularization was from $10^{-6}$ to 10 with $N=16$ logarithmically spaced values.

For the implementation of DP, the selected $\lambda _{DP}$ is determined by the following criterion^2^:

15 $$\begin{aligned} \text {Choose }\lambda = \lambda _{DP} \text { such that } \left\Vert \mathbf {A}\mathbf {f}_{\lambda _{DP}} - \mathbf {y}_{\text {ob}}\right\Vert _2 = \nu _{DP} \left\Vert {\varvec{\omega }}\right\Vert _2, \end{aligned}$$

where $\nu _{DP} \ge 1$ and $\left\Vert {\varvec{\omega }}\right\Vert _2 \approx \sqrt{m}\sigma ({\varvec{\omega }})$. We chose the safety factor $\nu _{DP}$ to be $\nu _{DP}=1.05$.

Figure 1 shows the comparison of SpanReg with the DP for recovery of the indicated underlying DF for a single noise realization. As seen, SpanReg exhibits greater ability to resolve the two components of the DF and accurately model their amplitudes.

**Figure 2:**
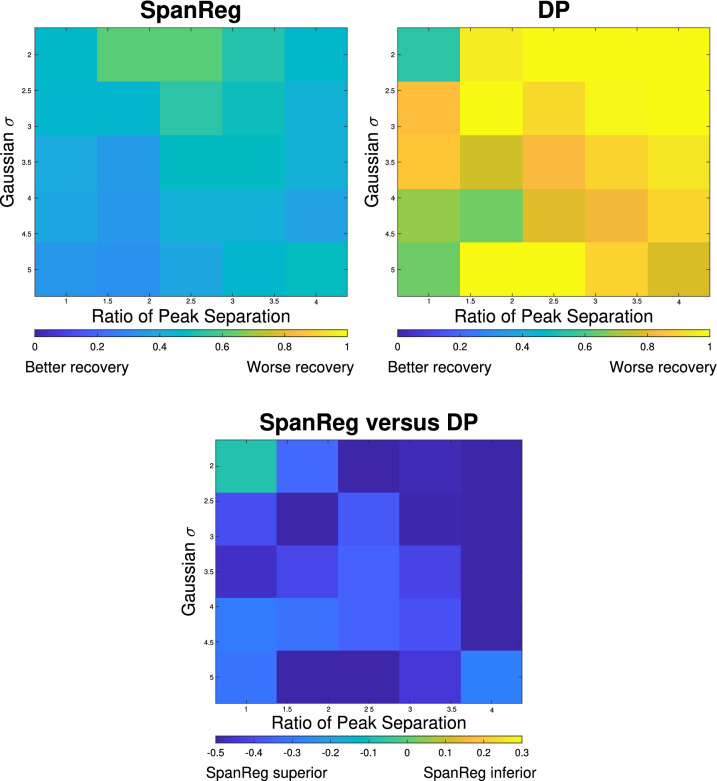
Heat maps showing relative errors for SpanReg (upper-left), discrepancy principle (upper-right), and their differences $\varepsilon _{\text {diff}}$ (bottom), as defined in the text.

Figure 2 presents the comparison of the two reconstruction methods in terms of heat maps of mean relative error, defined respectively for SpanReg and the DP by

16 $$\begin{aligned} \varepsilon _{\text {SpanReg}} = \frac{\left\Vert \mathbf {f}^*_{\text {SpanReg}} - \mathbf {f}_{\text {sim}}\right\Vert _2}{\left\Vert \mathbf {f}_{\text {sim}}\right\Vert _2},\, \varepsilon _{\text {DP}} = \frac{\left\Vert \mathbf {f}^*_{\text {DP}} - \mathbf {f}_{\text {sim}}\right\Vert _2}{\left\Vert \mathbf {f}_{\text {sim}}\right\Vert _2} \text{ and } \varepsilon _{\text {diff}} = \varepsilon _{\text {SpanReg}} - \varepsilon _{\text {DP}}, \end{aligned}$$

where $\mathbf {f}^*$ is the reconstructed distribution and $\mathbf {f}_{\text {sim}}$ is defined in Eq. (14). Thus, in terms of relative errors, a negative $\varepsilon _{\text {diff}}$ indicates the superiority of SpanReg reconstruction. We see that by this summary metric of relative error, SpanReg outperforms DP across all DF’s studied. Indeed, the errors for SpanReg are fairly constant across distributions, while the errors for DP increase as RPS increases over the illustrated range. Thus, as the component centers deviate from each other, including when closely-spaced, SpanReg is more able to accurately resolve them. In addition, both methods tend to perform better as the widths of the Gaussian distributions $\sigma$ of the target DF increase; this highlights the smoothing characteristics of the $L_2$ norm.

Additional insights are provided by comparing the heat map for the relative error metric described in Eq. (16), of use in summarizing the quality of reconstructions across a wide range of target DFs, with Fig. 1. From the latter we see that for closely-spaced Gaussian components, represented by the second column of Fig. 1, the reconstructions are not only quantitatively, but also qualitatively, different. The DP reconstruction incorrectly provides a single-component reconstruction in a number of cases, while SpanReg is clearly able to resolve the two underlying components. Therefore, it is important to examine the actual recovered $T_2$ distributions in addition to comparing the single summary metric defined by relative error.

**Figure 3:**
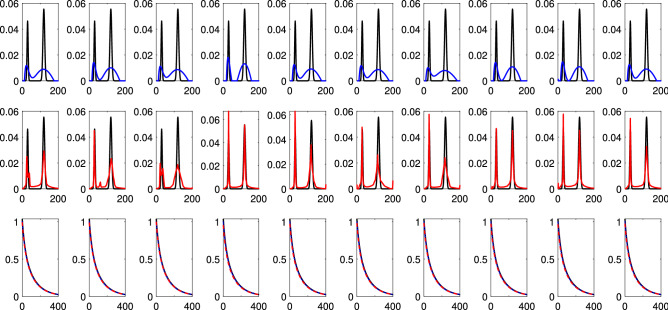
Reconstruction of the sum of two Gaussians with $(\mu _1, \sigma _1$) = (30 ms, 3 ms) and $(\mu _2, \sigma _2)$ = (120 ms, 5 ms), shown in black in the top and middle rows; SNR = 500. Each column corresponds to a single noise realization. Top: DP (blue), Middle: SpanReg (red), Bottom: signals generated from the DP solution and the SpanReg solution.

**Figure 4:**
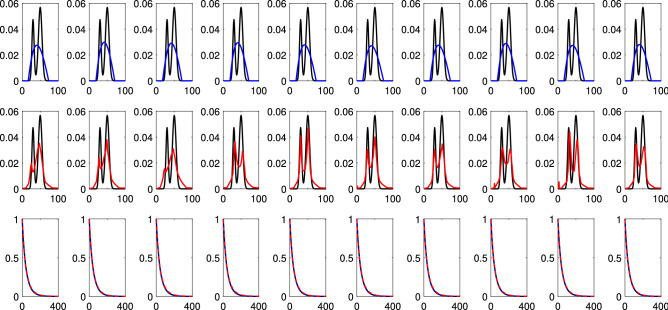
Reconstruction of the sum of two Gaussians with $(\mu _1, \sigma _1$) = (30 ms, 3 ms) and $(\mu _2, \sigma _2)$ = (50 ms, 5 ms), shown in black in the top and middle rows; SNR = 500. Each column corresponds to a single noise realization. Top: DP (blue), Middle: SpanReg (red), Bottom: signals generated from the DP solution and the SpanReg solution.

This is further highlighted in Figs. 3 and 4, comparing SpanReg and DP on two target distributions with respectively a greater and a lesser RPS. We show the results of reconstructions over 10 noise realizations, as well as a comparisons of the corresponding signals generated by these reconstructions. In Fig. 3, SpanReg, as compared to the DP, provides a much more accurate reconstruction of the two components in the DF, although both methods accurately recover two resolved components. In the much more ill-posed problem illustrated in Fig. 4, SpanReg clearly outperforms the DP in terms of stability with respect to noise and ability to resolve two closely-spaced peaks. DP fails to do this for all of the 10 noise realizations, while SpanReg succeeds in 7 out of 10 cases. Decreasing the DP safety factor improves the resolution performance of the DP, but introduces spurious peaks and greater noise sensitivity.

### Stability with respect to choice of regularization parameters

Selection of the optimal regularization parameter $\lambda$ is of critical importance in Tikhonov regularization, with deviations from that optimal value potentially resulting in substantial differences in the reconstructed DF. Figure S1 from the Supplementary Information shows the solutions obtained from Tikhonov regularization and from SpanReg over different ranges of $\lambda$’s, including in all cases the optimal value, $\lambda _{\text {opt}}$. In this simulation with a known underlying distribution, $\lambda _{\text {opt}}$ is determined by the $L_2$ error metric describing the difference between the underlying DF and the reconstructed DF:

17 $$\begin{aligned} \lambda _{\text {opt}} = \mathop {\hbox {argmin}}_{\lambda } \left\{ \left\Vert \mathbf {f}_{\lambda } - \mathbf {f}_{\text {sim}}\right\Vert _2 \right\} . \end{aligned}$$

Once $\lambda _{opt}$ is determined, stability is quantified by comparing the DF recovered using the different values of $\lambda$ from the set ${\mathscr {S}}=\left\{ \frac{\lambda _{\text {opt}}}{2^4}, \ldots ,\frac{\lambda _{\text {opt}}}{2^{1}},\frac{\lambda _{\text {opt}}}{2^0}, \frac{\lambda _{\text {opt}}}{2^{-1}},\ldots ,\frac{\lambda _{\text {opt}}}{2^{-5}}\right\}$. To apply this analysis to the DP, we compare solutions obtained with the different values of $\lambda$. We also show the differences $\Delta \mathbf {f}_{\frac{\lambda _{\text {opt}}}{2^j}} =\mathbf {f}_{\lambda _{\text {opt}}} - \mathbf {f}_{\frac{\lambda _{\text {opt}}}{2^j}}$ between the results for these $\lambda$’s and the result for $\mathbf {f}_{\lambda _{\text {opt}}}$; larger values in these difference plots indicate less stability. For SpanReg, we perform the analysis using different triads of adjacent values of $\lambda$ from ${\mathscr {S}}$. We form the reconstruction using the three $\lambda$’s centered around $\lambda _{\text {opt}}$, denoted $\mathbf {f}_{\text {SpanReg},\lambda _{\text {opt}}}$:

18 $$\begin{aligned} \mathbf {f}_{\text {SpanReg},\lambda _{\text {opt}}} := \alpha _1 \mathbf {f}_{\frac{\lambda _{\text {opt}}}{2}} + \alpha _2\mathbf {f}_{\lambda _{\text {opt}}} + \alpha _3 \mathbf {f}_{\frac{\lambda _{\text {opt}}}{2^{-1}}}, \end{aligned}$$

where the values $\left\{ \alpha _1, \alpha _2,\alpha _3\right\}$ are obtained from SpanReg. Then, to evaluate stability with respect to choice of $\lambda$, we plot results for SpanReg using three shifted values of $\lambda$’s. For example, for an integer $-3\le j\le 4$,

$$\begin{aligned} \mathbf {f}_{\text {SpanReg}, \frac{\lambda _{\text {opt}}}{2^j}} := \alpha _1 \mathbf {f}_{\text {SpanReg}, \frac{\lambda _{\text {opt}}}{2^{j+1}}} + \alpha _2\mathbf {f}_{\text {SpanReg}, \frac{\lambda _{\text {opt}}}{2^j}} + \alpha _3 \mathbf {f}_{\text {SpanReg}, \frac{\lambda _{\text {opt}}}{2^{j-1}}}. \end{aligned}$$

The comparison of the results for shifted triples of $\lambda$'s defines the stability of SpanReg with respect to choice of regularization parameter. We also plot the difference between the DF recovered by adjacent shifted triplets to the recovery obtained using the triplet centered on $\mathbf {f}_{\lambda _{\text {opt}}}$, i.e. we define $\Delta \mathbf {f}_{\text {SpanReg},\frac{\lambda _{\text {opt}}}{2^j}} = \mathbf {f}_{\text {SpanReg},\lambda _{\text {opt}}} - \mathbf {f}_{\text {SpanReg},\frac{\lambda _{\text {opt}}}{2^j}}$. These plots quantify the changes in the recovered DF resulting from a suboptimal selection of $\lambda$ in SpanReg. A larger magnitude in these difference plots corresponds to lower stability with respect to choice of $\lambda$ and conversely.

An example of this is shown in the Supplementary Information, Fig. S1, where results for the illustrated two-component Gaussian DF are shown for optimally regularized DP solutions and for solutions obtained with sub-optimal regularization (left panels). Corresponding solutions obtained with SpanReg using different sub-sequences of regularized solutions are also shown (right panels). Departures from the optimal regularization solutions obtained for Tikhonov regularization (lower left panel) are substantially larger than those using SpanReg (lower right panel). The corresponding $L_2$ norms of the differences are

$$\begin{aligned} \left( \left\Vert \Delta \mathbf {f}_{\lambda _1}\right\Vert , \left\Vert \Delta \mathbf {f}_{\lambda _2}\right\Vert , \left\Vert \Delta \mathbf {f}_{\lambda _4}\right\Vert , \left\Vert \Delta \mathbf {f}_{\lambda _5}\right\Vert \right) = \left( 0.23, 0.11, 0.08, 0.13 \right) \end{aligned}$$

for Tikhonov regularization and

$$\begin{aligned} \left( \left\Vert \Delta \mathbf {f}_{\text {SpanReg}, \lambda _1}\right\Vert , \left\Vert \Delta \mathbf {f}_{\text {SpanReg}, \lambda _2}\right\Vert , \left\Vert \Delta \mathbf {f}_{\text {SpanReg}, \lambda _4}\right\Vert , \left\Vert \Delta \mathbf {f}_{\text {SpanReg}, \lambda _5}\right\Vert \right) = \left( 0.14, 0.06, 0.05, 0.08 \right) \end{aligned}$$

for SpanReg, respectively, indicating a roughly 60$\%$ improvement in stability using SpanReg as compared to the DP.

### Myelin water fraction mapping of the human brain

Having shown the effectiveness of SpanReg on a wide range of simulated data, we now demonstrate its application to brain imaging. In particular, we compare the results of SpanReg, NNLS regularization using the DP, and non-regularized NNLS for conventional myelin water fraction (MWF) mapping in the human brain. In brief, this method involves acquiring a decaying bi-exponential signal for each image pixel, from which a DF of spin-lattice relaxation times $T_2$ is recovered. Given the high SNR of these images, we restrict our attention to the Gaussian noise approximation. Note again that the simulations above were targeted to parameter ranges appropriate for this problem of in vivo myelin mapping.

While the ground truth for brain MWF in vivo is unknown, we can still implement numerical experiments using this data to compare different reconstruction approaches. First, we defined the MWF as the integral of the $T_2$ DF between abscissa values of $T_2=6$ ms and $T_2= 40$ ms^15,20^ for each pixel and created the corresponding map. To compare two different MWF maps $\mathbf {A}$ and $\mathbf {B}$, we define the scaled absolute difference (SAD) by:

19 $$\begin{aligned} SAD(\mathbf {A}, \mathbf {B}) = \frac{S_{\mathbf {A}- \mathbf {B}}}{S_{\mathbf {A}}}, \end{aligned}$$

where $S_{\mathbf {M}} = \sum _{i,j} \left| M_{i,j}\right|$ is the sum of the absolute values of the elements of a matrix $M_{i,j}$. In this way, if $SAD(\mathbf {A},\mathbf {B}_1)$, as a comparison between a reference map $\mathbf {A}$ and an approximation $\mathbf {B}_1$, is smaller than $SAD(\mathbf {A},\mathbf {B}_2)$, we say that the reconstructed MWF map $\mathbf {B}_1$ exhibits superior accuracy to $\mathbf {B}_2$.

Because absolute magnetic resonance signal amplitudes are arbitrarily scaled by various sample characteristics, experimental settings and instrument properties, it is necessary to normalize the data so that $\int f(T_2) dT_2 = 1$ before applying SpanReg. This represents normalization of the PDF represented by the DF $f(T_2)$. Then, from Eq. (4) written in terms of *TE*, the signal must be normalized to its initial value, that is $y(TE=0)=1$. This is problematic for two reasons. First, for experimental reasons, the spin-echo experiment in particular has measurement times at $i \times TE$ with $i=1$ rather than $i=0$. This limitation does not apply to other similarly-modeled experiments, such as diffusion-sensitizing pulse sequences. In addition, however, in any experiment, all data points are corrupted by noise so that normalization of the acquired signal by its initial value is not equivalent to normalizing the underlying signal, which is what is desired; the underlying and observed signals differ by noise at each data point. Lacking a measured signal value at TE = 0 for normalization, we approximate it from what would have been the TE = 0 value, given the recovered distribution. This value is equal to $\left\Vert \mathbf {f}_{LS}\right\Vert _1$ according to the following:

20 $$\begin{aligned} \begin{aligned} y_{LS}\left( TE = 0\right)&\approx \left( e^{-0/T_{2,1}}, e^{-0/T_{2,2}},\cdots ,e^{-0/T_{2,n}}\right) \left( f_{LS,1}, f_{LS,2},\cdots ,f_{LS,n} \right) ^T \Delta T_2\\&= \sum _{j = 1}^n f_{LS,j} \Delta T_2 = \left\Vert \mathbf {f}_{LS}\right\Vert _1 \Delta T_2, \end{aligned} \end{aligned}$$

where the NNLS estimate of $\mathbf {f}_{LS}\ge \mathbf {0}$ is determined from non-normalized data according to Eq. (7).

We note that in effect, Eq. (7) is equivalent to Eq. (5), which is the original problem. In the current context, we are not representing the solution to Eq. (7) to be an accurate estimate of the entire DF $\mathbf {f}$, but rather as the best available estimate of $f_{TE = 0}$. This approach introduces no additional bias though use of regularization. In our imaging analyses, the observed data $\mathbf {y}_{\text {ob}}$ is divided by $\left\Vert \mathbf {f}_{LS}\right\Vert _1$ for each pixel. This analysis was not required for the simulation experiments above, where we illustrated SpanReg in the more usual case of signals with an initial abscissa value of zero. In the “Results” section, we implement this approach to reconstruct SpanReg-based MWF maps.

We present two distinct analyses for the brain dataset. First, we define a high-quality reference MWF map, based on pixel-wise reconstructions from SpanReg on NESMA-filtered data^20^. This serves as a standard of comparison for three reconstruction methods, SpanReg and NNLS with DP regularization and without regularization, for two different levels of SNR. Then, we work directly with NESMA-filtered (high SNR) and non-filtered (low SNR) images using all three reconstruction methods to demonstrate their performance with respect to SNR; a comparison is made between results obtained at high and low SNR for each method separately.

#### Comparison of reconstruction methods with respect to a reference image

**Figure 5:**
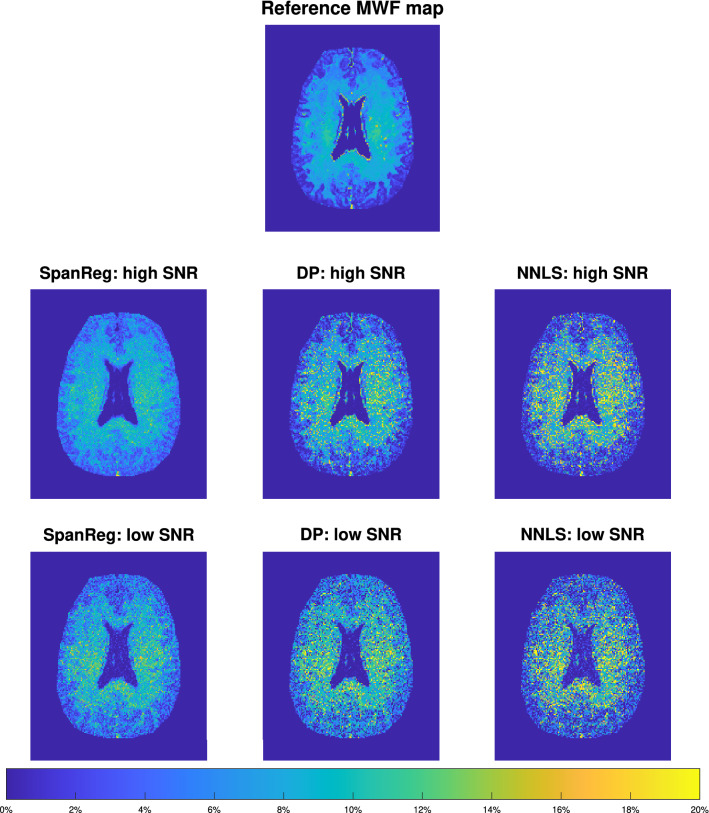
Uppermost image: reference MWF map; MWF maps generated from high SNR (top row; SNR = 800) and low SNR (bottom row; SNR = 200) data using SpanReg, NNLS with the DP, and non-regularized NNLS.

We obtained the reference MWF map shown in the uppermost panel of Fig. 5 as follows. First, we obtained a stack of successively $T_2$-weighted images from the data acquisition sequence as described above; this defines a decay curve corresponding to each pixel. However, before processing these curves as described in detail above, we first apply the NESMA filter to the stack of images, resulting in pixel-wise decay curves of greatly improved SNR. The decays are then inverted using SpanReg to obtain a $T_2$ distribution for each pixel. The reference MWF map is formed from these distribution as described above, by taking the integral of the DF’s up to $T_2$  = 40 ms for each pixel separately.

The MWF maps created from high and low SNR data for comparison, shown in the same figure, were generated as follows. The high-quality $T_2$ distribution functions used to define the reference map were used to generate noiseless decay curves, to which Gaussian noise was added to achieve SNR = 800 (high SNR) and SNR = 200 (low SNR) in the decays. The decay curve at each pixel corresponds to $\mathbf {y}_{\text {ob}}$ in Eq. (5). We then reconstructed the $T_2$ DF’s using SpanReg, and NNLS with DP regularization and without regularization. MWF maps were constructed from these and compared to the reference map using the SAD metric, Eq. (19).

Visual inspection shows that the SpanReg analysis yields a result more closely resembling the reference image than either of the other two methods. Quantitatively, the scaled absolute difference (SAD) between the reference map and the derived MWF maps from the high SNR dataset are: 0.32 (compared to SpanReg), 0.38 (NNLS with DP regularization), and 0.59 (non-regularized NNLS). The corresponding values for the low SNR dataset are: 0.39 (SpanReg), 0.58 (DP regularization), and 0.68 (non-regularized). Thus, by this metric, SpanReg outperforms DP for the high SNR and low SNR datasets by $16\%$ resp. $33\%$, and NNLS by $46\%$ resp. $43\%$.

#### Comparison of performance for high versus low SNR data

We now examine the sensitivity to noise of the reconstruction methods. Even with spatially invariant noise, SNR varies across an image because of signal strength variations. Ideally the entire SpanReg algorithm would be applied separately for each pixel according to its SNR. This is not practical for typical images; in our case, the images consist of $\sim 25{,}000$ pixels. Therefore, we divided the full range of image SNR into 18 bins over the range of 10 to 800, encompassing both unfiltered and NESMA-filtered pixel-wise decay signals. The same Gaussian basis functions were used in all cases, but different SpanReg parameter values and functions $\left\{ \beta _{i,\lambda _j}\right\}$, $\left\{ \mathbf {g}_{i,\lambda _j}\right\}$ were obtained for each of these 18 SNR bins based on its SNR. The corresponding $\left\{ \beta _{i,\lambda _j}\right\}$ and $\left\{ \mathbf {g}_{i,\lambda _j}\right\}$ were used to reconstruct the data from each pixel with SNR within that bin. MWF for each pixel was then derived from the resulting distributions as described above, by integrating the DF’s up to 40 ms. The resulting MWF maps are displayed in the Supplementary Information, Fig. S2.

We first note that the results for SpanReg are visually more similar at the two noise levels than are the results for the other reconstruction methods, indicating greater stability with respect to noise. In addition, the MWF distributions recovered by DP and LS show prominent edge effects surrounding the ventricles, as well as myelin voids towards the brain periphery. In contrast, the MWF maps obtained with SpanReg show a much more qualitatively normative pattern. Quantitatively, the SAD between the high and low SNR MWF maps, $SAD\left( \mathbf {M}_{\text {high SNR}} , \mathbf {M}_{\text {low SNR}} \right)$, for the three reconstruction methods are 0.35 for SpanReg, 0.43 for DP, and 0.63 for NNLS. This is consistent with the results shown in Fig. 5.

## Discussion

In this paper, we present a new approach, SpanReg, to obtain a regularized solution of the discretized Fredholm equation of the first kind. The analysis followed directly from the Fredholm equation of the first kind, so that we expect this technique to be widely applicable to a large category of inverse problems requiring regularization. The idea of the method is to incorporate information content over a range of regularized solutions rather than to attempt to identify a single optimal regularization parameter, $\lambda$. For inverse problems of a type arising extensively in MR relaxometry, we provided simulations demonstrating greater accuracy of SpanReg as compared to conventional Tikhonov regularization using the DP for selection of $\lambda$. We also showed that SpanReg is more stable with respect to the choice of regularization parameters, in the sense that the departure from an optimal solution is smaller when the solution arrived at using the optimal $\lambda$ is not incorporated. The method was also demonstrated on experimental biomedical MRR data to evaluate the MWF in the human brain; this is a very challenging problem of great ongoing interest^21,22^.

The framework put forward in this work is to provide a more general form of the recovered DF: $\mathbf {f}^* = \sum _j \alpha _j \mathbf {f}_{\lambda _j}$; conventional Tikhonov regularization incorporates just a single term, optimal in some sense, of this sum. In addition to relaxing the restriction of isolating the single best regularized solution, our method also provides a flexible form for the construction of the desired solution. While stabilized global features of the myelin water fraction (MWF) distribution are evident at high regularization levels, more detailed features of the recovered distributions require lower levels of regularization. This example motivates the idea of aggregating a range of regularized solutions; we do this by calculating the effect of noise on the representing dictionary functions. Through this we were able to capture the underlying features of the DF while preserving the stability of the recovery and avoiding the risk that a conventional selection of a single regularization parameter may produce a non-optimal solution. To obtain the coefficients $\left\{ \alpha _j\right\}$, we formulated the solution $\sum _j \alpha _j \mathbf {f}_{\lambda _j}$ as a linear combination of multiple regularized solutions via a dictionary of Gaussian functions $\left\{ \mathbf {g}_{i,\lambda _j} \right\}$. The algorithm can be separated into offline and online computations, where the offline part is specific to a given SNR and basis set, but is independent of a specific instance of experimental data. The offline calculation requires the determination of the noise-corrupted basis functions and parameters, e.g. fixing the number *M* of Gaussian dictionary functions $\mathbf {g}_{i}$, the number *N* of regularization parameters used by the algorithm, and performing the actual computation required to obtain the noise-corrupted DF, $\left\{ \mathbf {g}_{i,\lambda _j}\right\}$.The online calculation is dependent upon a specific data set and is much less computation-intensive, compared with online computations.

This formulation exhibits a condition number $cond(\mathbf {B}) = O(10^5)$ in Eq. (30) for the simulation analysis in Fig. 1, compared to the condition number of $cond(\mathbf {A})$ in the NNLS problem, which is $O(10^{20})$.

In this study, we demonstrated that the SpanReg method achieves higher accuracy than the conventional DP parameter selection method, which also requires knowledge of the noise level in the observation $\mathbf {y}_{\text {ob}}$. For the prototypical bimodal DF’s studies, SpanReg exhibits a substantially greater ability to resolve the two components. With more disparate spaced components, SpanReg also achieved improved reconstruction of positions and amplitudes of the components. Further, SpanReg is not constrained to the selection of a single optimal value of $\lambda$; in fact, in conventional practice, this optimal value will depend upon the specific selection criterion implemented. Thus, application of different conventional methods is virtually guaranteed to recover different DF’s. In contrast, our more general formulation forms the reconstruction through a linear combination of differently-weighted solutions with appropriate weights, and so is less dependent upon the exact degree of regularization of any one of these.

From the statistical perspective, the approach of linearly combining, or aggregating, different regularized solutions has been proposed and studied in the context of regression problems^23^. In addition, Chen et al.^24^ proposed the notion of aggregation of regularized solution in the context of inverse problems. Our approach to combining regularized solutions is different from the one proposed by Chen et al.,which is based on a particular implementation of the balance principle; see Eqs. (23) and (27) in Chen et al.^24^ Previous studies have also presented important reconstruction methods based on Tikhonov regularization with multiple regularization parameters. In distinct contrast to our work and that of Chen et al.^24^, these have employed a modified regularization penalty from which is derived a single optimal solution. These methods have been designated multiple regularization^25,26^, multi-parameter regularization^27–32^, and multi-penalty regularization^33,34^. This literature generally studies the same optimization problem as we have worked with in the general form:

21 $$\begin{aligned} \mathbf {f}^* = \mathop {\hbox {argmin}}_{\mathbf {f}}\left\{ \left\Vert \mathbf {y}_{\text {ob}}- \mathbf {A}\mathbf {f}\right\Vert _2^2 + \sum _{j = 1}^N \lambda _j {\mathscr {L}}_j\left( \mathbf {f}\right) \right\} , \end{aligned}$$

where ${\mathscr {L}}_j$ are regularization operators and $\lambda _j$ are regularization parameters associated with the corresponding operator. One of the most well-known of these is elastic-net regularization (EN)^13^, where ${\mathscr {L}}_1\left( \mathbf {f}\right) = \left\Vert \mathbf {f}\right\Vert _1$, and ${\mathscr {L}}_2\left( \mathbf {f}\right) = \left\Vert \mathbf {f}\right\Vert _2^2$. The EN is able to promote sparsity while also preserving accuracy for smoother distributions. Other types of penalty terms involving smoothness constraints have also been proposed; these include penalties of the form $\left\Vert \nabla \mathbf {f}\right\Vert _1$^25,26,29–32^, $\left\Vert \nabla \mathbf {f}\right\Vert _2^2$^29,31,33^ Other penalties of the form $\left\Vert \mathbf {W}\mathbf {f}\right\Vert$^25,26,28,32^ have been implemented, where $\mathbf {W}$ may be, for example a projection or transformation matrix. Unlike SpanReg, all of these methods seek optimal regularization parameters for a specific form of regularization rather than incorporating differently-regularized solutions.

An interesting alternative method more closely related to ours deviates from the formalism of Eq. (21) by constructing a linear combination of regularized solutions^35^, as in our treatment. The components forming the recovered DF are obtained from different regularization methods, with, as in conventional treatments and distinct from SpanReg, a single optimal regularizer being identified for each. Nevertheless, this work introduces the idea of combining DF’s, each of which in effect reflects a potentially desirable quality, into a final derived DF.

A variety of parameter selection techniques have been presented for the conventional types of multi-parameter regularization described above, generally based on the classical approaches for single-parameter regularization optimization. These include the multi-parameter discrepancy principle^29^, L-hypersurface^25,36^, and GCV-multi^32^. Other methods^28^ such as simple grid search^4^ and the balancing principle^34^ have been implemented. However, as emphasized above, none of these studies present the notion of including sub-optimal regularized solutions as we have proposed and demonstrated with SpanReg. Thus, the current work is fundamentally different from what has appeared in the literature in; we form a linear combination of sub-optimal solutions, in contrast to previous work which has used combinations of regularization terms leading to a single “optimal” solution, or^35^ a linear combination of optimal solutions. SpanReg allows us to combine the stability of more highly-regularized solutions with the resolution of less-regularized solutions; the potential for the efficacy of this construction was provided by our observations of the different responses to regularization of very similar ill-posed problems.

SpanReg should be widely applicable to a wide range of linear inverse problems based on the Fredholm equation of the first kind. As one class of examples, we demonstrated its applicability to MRR investigations of brain, where the DF is based on the $T_2$ distribution of tissue. However, the same formulation also applies to diffusion MRI^37^, where application is simpler due to the availability of the $b=0$ data point, where *b* is the conventional symbol for the combination of gradient strengths and timing that defines diffusion sensitization. In this case, the formalism of Eq. (20) is not required. Similar comments apply to the closely-related problem of $T_1$ MRR^38^. Further, MRR applications extend far beyond those in biomedicine, and include the food sciences^39^, engineering^40^, and the petrochemical industry^41^. There are also many potential applications of SpanReg outside of MR studies, including fluorescence analysis^42^.

Although we have presented a novel and effective method for combining multiple degrees of regularization into an ill-conditioned problem, certain limitations remain. We have not fully explored the selection of an optimal basis, although the Gaussian basis set used has been shown to perform well for reconstructing a range of challenging DF’s. In addition, there are a number of user-selected parameters for SpanReg, and we have not yet explored these systematically. These include the number *N* of regularized solutions to incorporate into the DF as well as their associated regularization parameter values $\lambda _j$; the size *M* and details of the Gaussian dictionary used to represent the DF for each of these solutions; and the number $n_{\text {run}}$ of noise realizations used for averaging to render the results robust with respect to noise realization. In this paper, our considerations have been based on the goal of maintaining reasonable conditioning of the the LS problem in Eq. (23); as demonstrated, this empirical approach has worked well. We also emphasize that the requirement for knowledge of SNR is a limitation of SpanReg in many contexts, although for the large category of problems based on decaying signals, including MRR and related experiments in MR, SNR estimates are generally available. This consideration also applies to certain conventional parameter selection methods such as DP. In spite of these limitation, we have shown that SpanReg can be applied to datasets with varying, though known, levels of SNR, such as in the brain MWF analysis. This does require a lengthier offline computation, with separate calculations required across a range of SNR values. The degree to which a given range of SNR values must be discretized also remains an open question, though the success of the binning procedure described in the brain MRI analysis supports the notion that this discretization need not be unduly fine. This is a critical finding, indicating that the noise estimate required for SpanReg can be approximate.

In addition to theoretical developments and demonstration on simulated data, we have provided an analysis of in vivo brain MRI data. We compare the performance of SpanReg to two other methods for recovering DF’s from decay curves for application to brain MWF mapping. This presentation is not intended to provide an extensive comparison of SpanReg with other state-of-the-art methods for assessing MWF; development of these methods is a topic of major current interest, with rapid introduction of new approaches^20,43,44^. A full comparison with these is beyond the scope of the present manuscript, which is to introduce and provide an initial application of SpanReg.

In addition to addressing the current limitations of SpanReg as outlined above, potential extensions include exploration of alternative forms of Tikhonov and other types of regularization penalties. In addition, SpanReg can be extended in a straightforward fashion to 2D and higher dimensional MRR analysis^45^, for which the governing inverse problem is a higher-dimensional inverse Laplace transform.

In conclusion, we have proposed a new approach to determine regularized solutions to the Fredholm integral equation of the first kind by incorporating the information content of non-optimal solutions, and have demonstrated its efficacy in simulations and through application to MRR of the human brain. SpanReg should be widely applicable throughout the field of inverse problems, presenting an alternative to Tikhonov and related forms of regularization.

## Materials and methods

### Analytic framework

#### Theory

To link the two expressions (12) and (13), we define a symbolic inversion operator $\mathbf {A}^{-1}_{\lambda _j}$ as follows: given a vector of noisy observations $\mathbf {y}_{\text {ob}}$, $\mathbf {A}^{-1}_{\lambda _j}\mathbf {y}_{\text {ob}}:=\mathbf {f}_{\lambda _j}$, where $\mathbf {f}_{\lambda _j}$ is the solution of (8) with $\lambda =\lambda _j$, $j=1,\ldots ,N$. This operator is not the usual matrix pseudoinverse because of the non-negativity constraints.

We apply this to the noisy observations that would correspond to the signal generated by the $\mathbf {g}_i$, obtaining a set of associated DF’s $\{\mathbf {g}_{i,\lambda _j}\}$, $i=1,\ldots ,M$, $j=1,\ldots ,N$ according to Eq. (8):

22 $$\begin{aligned} \mathbf {g}_{i,\lambda _j} = \mathbf {A}^{-1}_{\lambda _j} \left( \mathbf {A}\mathbf {g}_i + {\varvec{\omega }}\right) , \end{aligned}$$

where ${\varvec{\omega }}$ represents a noise realization exhibiting the same noise level, defined by RMS($\omega$), as the observed data $\mathbf {y}_{\text {ob}}$. Note that while determining noise amplitude within an experimental signal can be highly problematic, in our examples of decaying exponentials this information is directly available from data collected from the signal tail.

We now seek to approximate both $\mathbf {f}_{\alpha }$ and $\mathbf {f}_{\mathbf {c}}$ by the $\{\mathbf {g}_{i,\lambda _j}\}$.

We first write

23 $$\begin{aligned} \mathbf {g}_i \approx \sum _{j = 1}^N \beta _{ij} \mathbf {g}_{i,\lambda _j}, \quad \beta _{ij}\ge 0. \end{aligned}$$

The expansion coefficients $\beta _{ij}$ would be Kronecker $\delta$’s in the absence of noise and regularization. As ${\varvec{\omega }}$ is random, the corresponding $\{\mathbf {g}_{i,\lambda _j}\}$ and $\{\beta _{ij}\}$ are random variables. Let $\langle \cdot \rangle$ denote the ensemble average of a random variable over $n_{\text {run}}$ noise realizations. In particular,

$$\begin{aligned} \langle \mathbf {g}_{i,\lambda _j}\rangle = \frac{1}{n_{\text {run}}} \sum _{k = 1}^{n_{\text {run}}} \mathbf {g}_{i,\lambda _j}^{(k)},\quad \langle \beta _{ij}\rangle = \frac{1}{n_{\text {run}}} \sum _{k = 1}^{n_{\text {run}}} \beta _{ij}^{(k)} \end{aligned}$$

where the superscript (*k*) indicates the *k*-th noise realization.

We now apply a representation analogous to Eq. (13) to define regularized approximations:

24 $$\begin{aligned} \mathbf {f}_{\lambda _j} \approx \sum _{i = 1}^M x_{ij} \langle \mathbf {g}_{i,\lambda _j} \rangle , \end{aligned}$$

where the $\{x_{ij}\}$ can be obtained through least squares analysis.

Thus, given the dictionary of Gaussian distributions $\left\{ \mathbf {g}_{i}\right\} _{i=1}^M$, we can find the coefficients $\left\{ \langle \beta _{ij}\rangle \right\}$ and $\left\{ x_{ij}\right\}$ from the approximations to the regularized solutions $\left\{ \mathbf {f}_{\lambda _j} \right\}$ and $\left\{ \langle \mathbf {g}_{i,\lambda _j}\rangle \right\}$, respectively. Now equating (12) and (13) and using (23) and (24), we arrive at an expression containing only $\{\langle \beta _{ij}\rangle \}$, $\{x_{ij}\}$ and $\{\langle \mathbf {g}_{i,\lambda _j}\rangle \}$:

25 $$\begin{aligned} &\sum _{j = 1}^N \alpha _j \mathbf {f}_{\lambda _j} \approx \sum _{i = 1}^M c_i \mathbf {g}_i\\&\quad \Rightarrow \mathbf {f}_{\alpha } := \sum _{j = 1}^N \alpha _j \sum _{i = 1}^M x_{ij} \langle \mathbf {g}_{i,\lambda _j}\rangle \approx \sum _{i = 1}^M c_i \sum _{j = 1}^N \langle \beta _{ij}\rangle \langle \mathbf {g}_{i,\lambda _j}\rangle =: \mathbf {f}_{\mathbf {c}}. \end{aligned}$$

The coefficients $\varvec{\alpha }$ and $\mathbf {c}$ are obtained by solving the least squares problem:

26 $$\begin{aligned} \left\{ \begin{aligned} &(\varvec{\alpha }^*,\mathbf {c}^*) = \mathop {\hbox {argmin}}\left\Vert \mathbf {f}_{\alpha } - \mathbf {f}_{\mathbf {c}}\right\Vert _2\\ & \text {subject to } \varvec{\alpha }^* \ge \mathbf {0},\; \mathbf {c}^*\ge \mathbf {0}, \text { and }\sum _i c^*_i = 1. \end{aligned} \right. \end{aligned}$$

Our expression for the final recovered $\mathbf {f}^*$ is then:

27 $$\begin{aligned} \mathbf {f}^*_{\alpha } = \sum _{j=1}^N \alpha _j{^*} \mathbf {f}_{\lambda _j}. \end{aligned}$$

Equation (27) is the main result of our analysis, defining the desired recovered DF in terms of a linear combination of differently-regularized solutions based on the observed data. Alternatively, the corresponding result for $\mathbf {f}^*_c$ may also be used with essentially equivalent results.

#### Numerical implementation

We define the following notation:

$$\begin{aligned} \mathbf {L}_{\alpha }&= \begin{pmatrix} \begin{pmatrix} \langle \mathbf {g}_{1,\lambda _1}\rangle&\langle \mathbf {g}_{2,\lambda _1}\rangle&\cdots&\langle \mathbf {g}_{M,\lambda _1}\rangle \end{pmatrix}&,\cdots ,&\begin{pmatrix} \langle \mathbf {g}_{1,\lambda _N}\rangle&\langle \mathbf {g}_{2,\lambda _N}\rangle&\cdots&\langle \mathbf {g}_{M,\lambda _N} \rangle \end{pmatrix} \end{pmatrix} \in \mathbb {R}^{n\times MN}\\ \mathbf {L}_{c}&= \begin{pmatrix} \begin{pmatrix} \langle \mathbf {g}_{1,\lambda _1}\rangle&\langle \mathbf {g}_{1,\lambda _2}\rangle&\cdots&\langle \mathbf {g}_{1,\lambda _N}\rangle \end{pmatrix}&,\cdots ,&\begin{pmatrix} \langle \mathbf {g}_{M,\lambda _1}\rangle&\langle \mathbf {g}_{M,\lambda _2}\rangle&\cdots&\langle \mathbf {g}_{M,\lambda _N}\rangle \end{pmatrix} \end{pmatrix}\in \mathbb {R}^{n\times MN}\\ \mathbf {x}_{\text {vec}}&= \begin{pmatrix} \begin{pmatrix} x_{11}&x_{21}&\cdots&x_{M1} \end{pmatrix}&,\cdots ,&\begin{pmatrix} x_{1N}&x_{2N}&\cdots&x_{MN} \end{pmatrix} \end{pmatrix}\in \mathbb {R}^{MN}\\ \varvec{\beta }_{\text {vec}}&= \begin{pmatrix} \begin{pmatrix} \langle \beta _{1,\lambda _1} \rangle&\langle \beta _{1,\lambda _2}\rangle&\cdots&\langle \beta _{1,\lambda _N}\rangle \end{pmatrix}&,\cdots ,&\begin{pmatrix} \langle \beta _{M,\lambda _1}\rangle&\langle \beta _{M,\lambda _2}\rangle&\cdots&\langle \beta _{M,\lambda _N}\rangle \end{pmatrix} \end{pmatrix}\in \mathbb {R}^{MN} \end{aligned}$$

where for each pair of fixed *i* and *j*, the regularized approximation $\langle \mathbf {g}_{i,\lambda _j}\rangle$ is a column vector of dimension $\mathbb {R}^n$ and the elements $x_{ij}$ and $\langle \beta _{i\lambda _j}\rangle$ are scalars. Thus, the lengths of the vectors $\mathbf {x}_{\text {vec}}$ and $\varvec{\beta }_{\text {vec}}$ are equal to the number of columns in $\mathbf {L}_{\alpha }$ and $\mathbf {L}_{c}$, respectively. Moreover, we write for the unknowns:

$$\begin{aligned} \varvec{\alpha }_{\text {vec}} & = \begin{pmatrix} \begin{pmatrix} \alpha _1&\alpha _1&\cdots&\alpha _1&\alpha _1 \end{pmatrix}_{M}&,\cdots ,&\begin{pmatrix} \alpha _N&\alpha _N&\cdots&\alpha _N&\alpha _N \end{pmatrix}_{M} \end{pmatrix}^T\in \mathbb {R}^{MN}\\ \mathbf {c}_{\text {vec}} & = \begin{pmatrix} \begin{pmatrix} c_1&c_1&\cdots&c_1&c_1 \end{pmatrix}_{N}&,\cdots ,&\begin{pmatrix} c_M&c_M&\cdots&c_M&c_M \end{pmatrix}_{N} \end{pmatrix}^T \in \mathbb {R}^{MN} \end{aligned}$$

Then Eqs. (12) and (13) can be written:

$$\begin{aligned} \mathbf {f}_{\alpha }&= \mathbf {L}_{\alpha }\cdot \text {diag}\left( \mathbf {x}_{\text {vec}} \right) \cdot \varvec{\alpha }_{\text {vec}} \\ \mathbf {f}_{c}&= \mathbf {L}_{c}\cdot \text {diag}\left( \varvec{\beta }_{\text {vec}} \right) \cdot \mathbf {c}_{\text {vec}} \end{aligned}$$

where $\cdot$ denotes the usual matrix-vector multiplication and $\text {diag}$ indicates the diagonal matrix formed from the vector argument. Equation (26) can then be re-formulated as:

28 $$\begin{aligned} \left\{ \begin{aligned} (\mathbf {c}^*,\varvec{\alpha }^*)&= \mathop {\hbox {argmin}}\left\Vert \mathbf {L}_{\alpha }\cdot \text {diag}\left( \mathbf {x}_{\text {vec}} \right) \cdot \varvec{\alpha }_{\text {vec}} - \mathbf {L}_{c}\cdot \text {diag}\left( \varvec{\beta }_{\text {vec}} \right) \cdot \mathbf {c}_{\text {vec}} \right\Vert _2\\&\text {subject to } \mathbf {c}\ge \mathbf {0},\varvec{\alpha } \ge \mathbf {0}, \sum _i c_i = 1 \end{aligned}\right. \end{aligned}$$

By writing the solution in the stacked form:

29 $$\begin{aligned} \mathbf {s}= \left( \alpha _1,\alpha _2,\cdots ,\alpha _{N},c_1,c_2,\cdots ,c_M \right) ^T \in \mathbb {R}^{N+M}, \end{aligned}$$

(28) can be expressed as a conventional LS problem for $\mathbf {s}^* \in \mathbb {R}^{N+ M}$

30 $$\begin{aligned} \left\{ \begin{aligned} \mathbf {s}^*&= \mathop {\hbox {argmin}}_{\mathbf {s}\ge \mathbf {0}} \left\Vert \mathbf {B}\mathbf {s}\right\Vert _2\\&\text {such that } \sum _{j=N+1}^{N+M} s_j = 1. \end{aligned}\right. \end{aligned}$$

with

$$\begin{aligned} \mathbf {B}&= \mathbf {L}_{\alpha }\cdot \text {diag}\left( \mathbf {x}_{\text {vec}} \right) \cdot \mathbf {TT}_{\alpha } - \mathbf {L}_{c}\cdot \text {diag}\left( \varvec{\beta }_{\text {vec}} \right) \cdot \mathbf {TT}_{c} \in \mathbb {R}^{n\times (N+M)}\\ \mathbf {TT}_{\alpha }&= \mathbf {I}_N \otimes \begin{pmatrix} 1\\ 1\\ \vdots \\ 1 \end{pmatrix}_M \begin{pmatrix} \mathbf {I}_{N}&\mathbf {0}_{ N \times M} \end{pmatrix} \in \mathbb {R}^{MN \times (N+M)}\\ \end{aligned}$$

and

$$\begin{aligned} \mathbf {TT}_{c} = \mathbf {I}_M \otimes \begin{pmatrix} 1\\ 1\\ \vdots \\ 1 \end{pmatrix}_N\begin{pmatrix} \mathbf {0}_{M \times N}&\mathbf {I}_{M} \end{pmatrix} \in \mathbb {R}^{MN \times (N+M)} \end{aligned}$$

where $\otimes$ is the Kronecker tensor product, $\mathbf {I}_M$, $\mathbf {I}_N$ are the identity matrices of rank *M* and *N*, respectively, and $\mathbf {0}_{N\times M}$ is the zero matrix of size $N\times M$.

The computational procedure can be divided into two parts, which we refer to as the offline part, meaning independent of the actual data set, and the data-dependent online part. In the offline computation, $\left\{ \mathbf {g}_i \right\}$, $\left\{ \langle \mathbf {g}_{i,\lambda _j}\rangle \right\}$ and $\left\{ \langle \beta _{i,\lambda _j}\rangle \right\}$ are determined only once. For the online part, for each noisy measurement $\mathbf {y}_{\text {ob}}$, the corresponding $\left\{ \mathbf {f}_{\lambda _j}\right\}$, $\left\{ x_{ij}\right\}$ and $(\mathbf {c}^*,\varvec{\alpha }^*)$ can be obtained with the desired solution given by (27). Note that the $\mathbf {g}_{i,\lambda _j}$, and hence the $\beta _{i,\lambda _j}$ in (23), are noise-dependent. For computations, we use the ensemble average over $n_{\text {run}}$ of realizations of Eq. (23).

The pseudocode for the offline and online computations reads as follows:
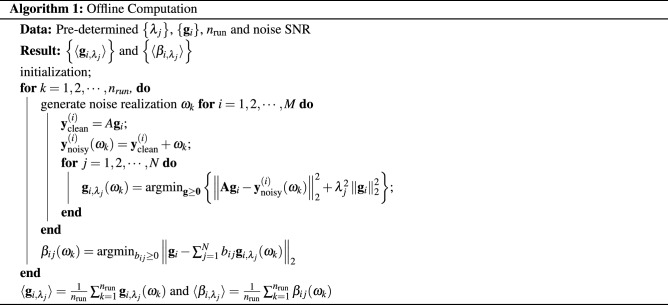

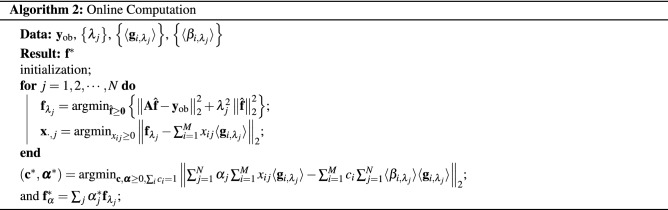


### Parameter settings for the implementation of SpanReg

Several user-defined parameters must be selected before proceeding with the off-line computation. We first fix the values of *N*, *M* and $n_{\text {run}}$, which are the number of regularization parameters, the number of Gaussian functions in the dictionary and the number of noise realizations, respectively. The values of $\lambda _j$, $j=1,\ldots ,N$ and the means and standard deviations (SD) of the Gaussian functions $\left\{ \mathbf {g}_i\right\} _{i=1}^M$ in the dictionary must also be selected.

The range of regularization parameters are determined based on L-curve analysis, with particular attention to parameters that provide a wide range of distinct solutions to Eq. (8). In the following simulations, we chose *N* = 16 with $\lambda _1,\cdots , \lambda _N$ logarithmically spaced over the interval $[10^{-6}, 10^1]$. The $T_2$ axis range is from 1 to 200 ms, discretized at 1 ms intervals, i.e. $\Delta T_2=1$. We chose a dictionary consisting of three families of Gaussians, each of which has its mean values equally spaced along the $T_2$ axis, and has a specified SD. The three families respectively consist of 160 members with SD = 2 ms, 40 members with SD = 3 ms, and 20 members with SD = 4 ms; $M = 160 + 40 + 20= 220$.

### Myelin water fraction mapping of the human brain

3D gradient and spin-echo (GRASE) images were obtained from the brain of a healthy 49-year-old female using 32 echoes at $TE_i= i\times TE$, where $TE = 11.3$ ms for $i = 1,2,\ldots , 32$. The notation *TE*, standing for echo time, is conventional in magnetic resonance, and corresponds to the measurement times in Eq. (4). The acquisition sequence had additional parameters of $TR = 1000$ ms, echo planar imaging acceleration factor of 3, field of view $278 \text { mm} \times 200\text { mm} \times 30\text { mm}$, acquisition matrix of size $185 \times 133 \times 10$, acquisition voxel size $= 1.5 \text { mm} \times 1.5\text { mm} \times 3\text { mm}$, reconstructed to $= 1\text { mm} \times 1\text { mm} \times 3\text { mm}$ using zero filling in k-space. Scan time was approximately 10 minutes. A 3T Philips MRI system (Achieva, Best, the Netherlands), equipped with an internal quadrature body coil for transmission and an eight-channel phased-array head coil for reception, was used for acquisition.

We employ both unfiltered and NESMA-filtered datasets. NESMA^20^ is a highly effective nonlocal denoising image filter that has been shown to increase the quality of myelin water fraction (MWF) mapping. The SNR of the unfiltered dataset is $\sim 10{-}300$, as defined by Eq. (6), with variation across pixels due primarily to the variation in image amplitude.

## Supplementary Information

Supplementary Figures.

## Data Availability

The dataset and code used and/or analyzed during the current study are available from https://doi.org/10.5281/zenodo.5860653.
