# Supplementary material for: Span of regularization for solution of inverse problems with application to magnetic resonance relaxometry of the brain
Source: Sci Rep. 2022 Nov 23;12:20194. doi: 10.1038/s41598-022-22739-3 (PMC9684479; doi:10.1038/s41598-022-22739-3)
Supplement: Supplementary file 1 — Supplementary Figures. [file 41598_2022_22739_MOESM1_ESM.pdf]

Supplementary information for

Span of regularization for solution of inverse problems with application to magnetic resonance relaxometry of the brain

Chuan Bi*, M. Yvonne Ou, Mustapha Bouhrara, Richard G. Spencer

*Corresponding author. E-mail: chuan.bi@som.umaryland.edu

This PDF file includes:

Figs. S1 to S2.

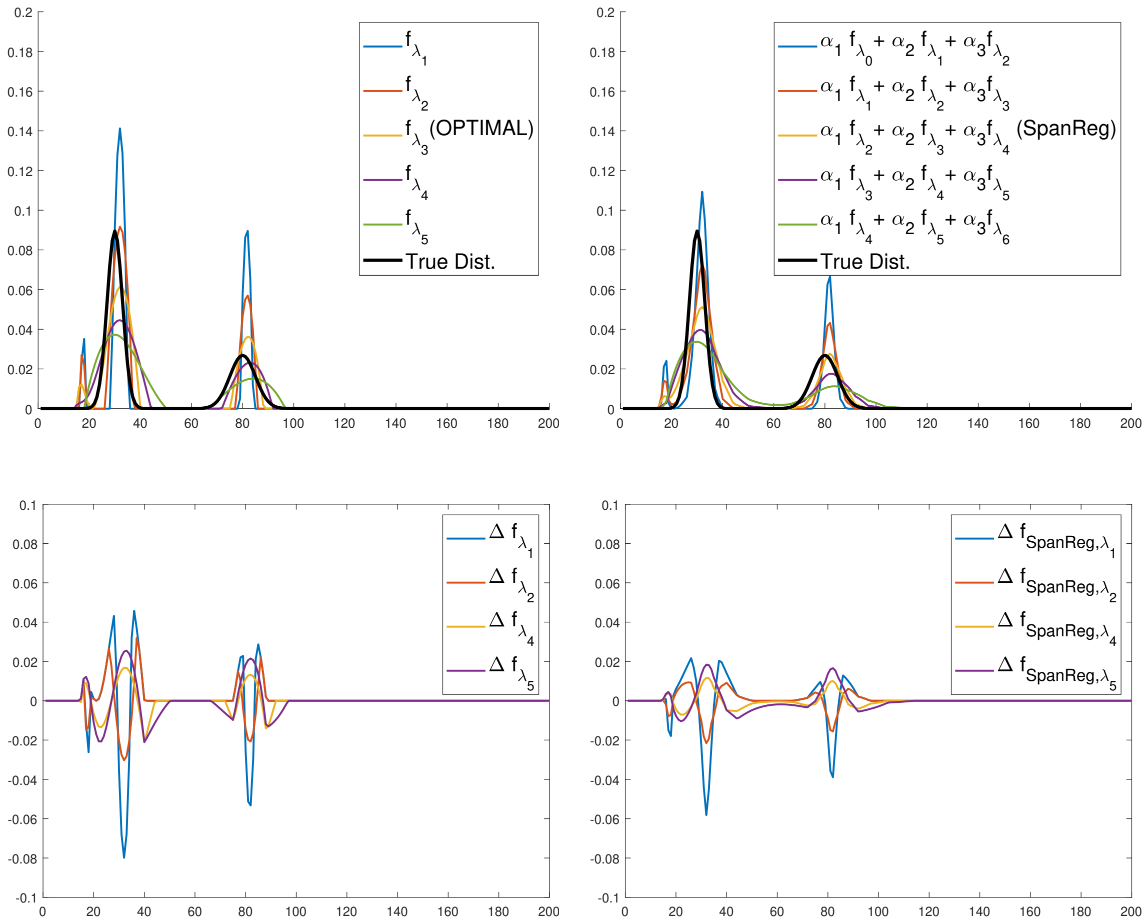

Figure S1: Stability with respect to choices of λ's, using a single value of λ for Tikhonov regularization and triplets of λ's for SpanReg. In all cases, SNR=500. The true distribution is $\frac{2}{3}g(30, 80) + \frac{1}{3}g(3, 5)$. The optimal solution is f_{λ_3}. Upper left: Solutions obtained with optimal and sub-optimal regularizations. Upper right: Solutions obtained with SpanReg with subsequences of regularized solutions. Bottom left: Departures from optimal regularized solution in the upper left panel. Bottom right: Departures from corresponding SpanReg solution in the upper right panel.

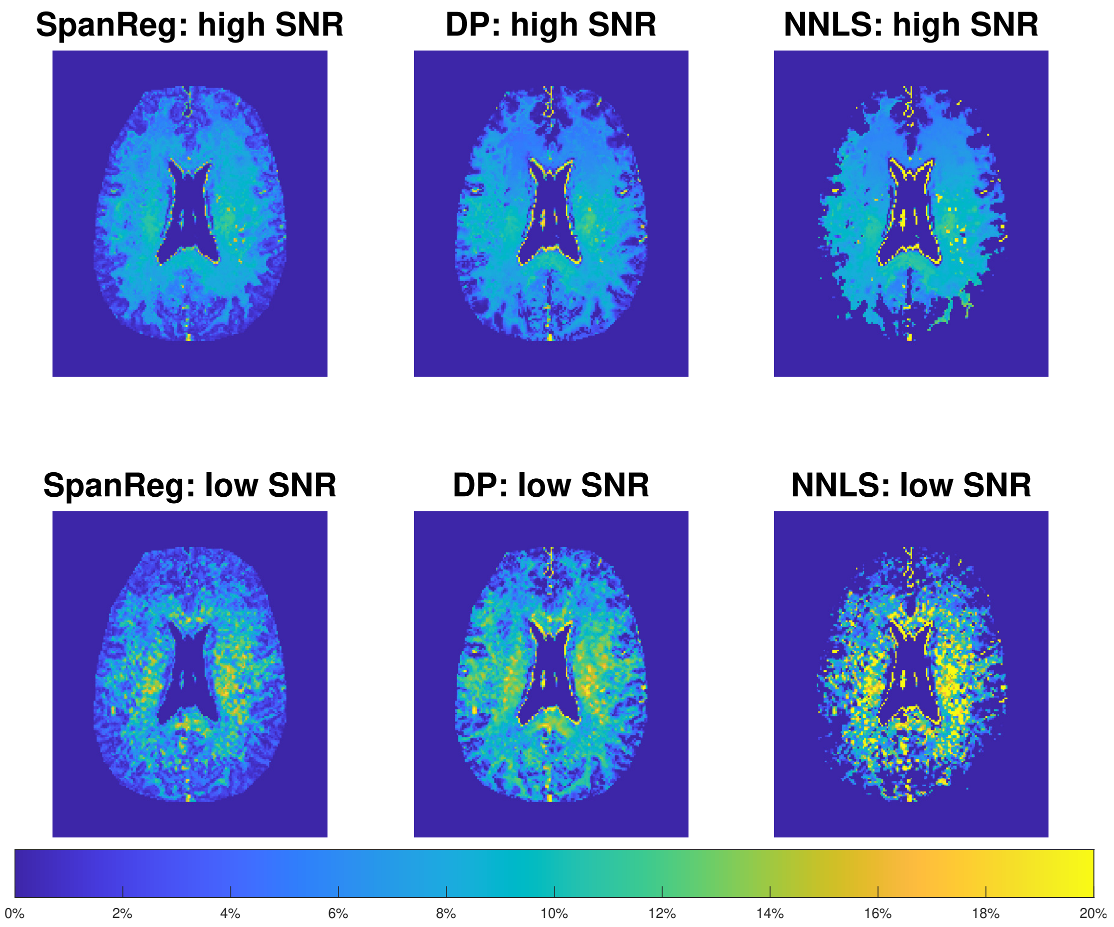

Figure S2: MWF maps reconstructed from original imaging data, to which two different levels of noise were added.
